# Risk Assessment of RYR Food Supplements: Perception vs. Reality

**DOI:** 10.3389/fnut.2021.792529

**Published:** 2021-12-07

**Authors:** Laura Righetti, Chiara Dall'Asta, Renato Bruni

**Affiliations:** Department of Food and Drug, University of Parma, Parma, Italy

**Keywords:** dietary supplement, botanical, mycotoxin, *Monascus*, quality control

## Abstract

Thirty-seven red yeast rice (RYR) food supplements were screened for their mycotoxin and natural statin content. Products included pure RYR capsules and multi-ingredient formulations with standardized amounts of monacolin K (MK), marketed both online and retail in the European Union. In terms of mycotoxins, citrinin (CIT) was found in all the monitored products. As CIT content ranged from 100 to 25100 μg/kg, only four products were compliant with maximum EU levels in force until April 2020, while a single product was compliant with the limit of 100 μg/kg introduced after that date. Four contaminated products were labeled as “citrinin free”. In terms of natural statins, nine products had a lower content vs. label statements (from −30 to −83%), while for 24 a larger MK amount (from 10 to 266%) was noticed. Three products had a negligible MK content and only 19 offered a daily dosage exceeding 10 mg as dictated by the health claim granted by EFSA in the EU. No sample had label values compliant with pharmaceutical Good Manufacturing Practices requirements (95–105% content of active constituent). Variable, but small amounts of simvastatin (0.1–7.5 μg per daily dose) were found in 30 samples. These results suggest that limited efficacy and reported safety issues may stem from an under-regulated and undercontrolled market, weakening both effectiveness and risk assessment evaluations.

## Introduction

Global sales of food supplements are growing steadily, sustained by consumers perceiving them as a safe and healthy option, mostly according to a supposed pharmaceutical quality in validation, manufacturing and control (1, 2). Quality control of these products is a multifaceted process, given the manifold traits and the variety of substances involved, whose complexity is often overlooked by the narrative surrounding them. For instance, in both the European Union and the United States, standards comparable to pharmaceutical Good Manufacturing Practices are not in force for the food supplement industry (3). Likely as a consequence, a variety of contamination and standardization concerns emerged during the last decade. Many supplements are in fact reportedly suffering from unreliable content of active principles, adulterations with foreign (and sometimes illicit or synthetic) substances, substitution with cheaper ingredients and pesticide or mycotoxin contamination (4–7). While these issues may emerge separately, they may also occur at the same time, altering trustworthiness of research data, safety of use and efficacy. To some extent, both conflicting results in clinical trials, difficult translations of scientific evidence into actual effectiveness, emergence of adverse effects and unreliability of most risk-benefit or risk assessment evaluations, may be a relapse of erratic quality of food supplements (3).

Multiple reports have repeatedly highlighted the lack of consistency between label and actual content in food supplement based on bilberry, blueberry, cranberry and ginkgo, to name a few (8–10). Furthermore, the issues summarized by Lockwood et al. in 2011 have not been corrected according to the subsequent literature, with an adulteration rate in herbal products exceeding 25% (11, 12).

Such scenario has prompted researchers to advocate for a closer attention to quality, for more widespread controls and for a stricter legal framework, to close the gap between reality and consumers perception in terms of quality and safety (13, 14).

Within this context, RYR represents a paradigmatic case. Following the demonstration that MK produced by *Monascus purpureus* grown on polished rice competitively inhibit human 3-hydroxy-3-methylglutaryl coenzyme A (HMG-CoA) reductase, a key enzyme in the biosynthesis of endogenous cholesterol, several studies confirmed the efficacy of RYR as a lipid-lowering agent. Its usefulness was demonstrated multiple times and is further reinforced by the structural equivalence between MK and lovastatin, the first statin made commercially available in the pharmaceutical market (15). After an extensive review, the European Food Safety Authority (EFSA) approved a specific health claim applicable to any RYR food supplement, stating that “Monacolin K from RYR contributes to the maintenance of normal blood cholesterol concentrations”. In order to obtain the claimed effect, 10 mg of MK from fermented RYR should be consumed daily. At the same time, despite the large commercial success, no specifications for RYR are available in European, Japanese, Canadian, US Pharmacopeias or in the WHO monographs.

The use of RYR is widespread, especially among the general population, in form of over-the-counter supplements produced by multiple brands and suggested as an alternative to prescription drugs. Besides the issue related to the homology between MK and Lovastatin, some criticality related to quality and safety emerged (16). Most relevant ones involve the erratic MK content, the emergence of side effects and the presence of citrinin, a polyketide mycotoxin with mutagenic, genotoxic and carcinogenic properties (17–20).

Citrinin is a nephrotoxic substance produced by few *Aspergillus* and *Penicillum* species, but is often detected also in *Monascus purpureus* cultures. It disrupts renal function leading to necrosis of the distal tubular epithelium, ultimately impairing renal function. It can be found in a wide variety of cereals, seeds and fruits, but its presence is rather common in food supplements based on RYR, to the point that a recent survey on the occurrence of citrinin in food commissioned by EFSA concluded that these supplements are the samples most prone to contamination with citrinin (21, 22).

EFSA also concluded that, “the composition and content of minor monacolins in food supplements containing fermented red rice is variable and the data does not allow to evaluate their contribution to the cholesterol-lowering efficacy” (23). As a consequence, the European Commission was recently persuaded to introduce a stricter limit for the presence of citrinin, whose permitted maximum amount in food supplements containing RYR was reduced in April 2020 from 2000 to 100 μg/kg (24, 25).

This study was therefore designed to estimate if RYR supplements available in the marketplace when new EU regulation became effective are compliant with the new limits. Further aims concerned the evaluation of concurrent quality issues, the level of compliance between declared and actual MK content and a detailed evaluation of the actual exposure to citrinin and monacolin K in terms of risk assessment.

## Materials and Methods

### Chemicals and Reagents

Citrinin (solution in acetonitrile 100 μg mL^−1^), ochratoxin A (solution in acetonitrile 100 μg mL^−1^) and aflatoxin mix containing AFB1 and AFB2 (2 μg mL^−1^ in acetonitrile), AFG1 and AFG2 (0.5 μg mL^−1^ in acetonitrile) were obtained from Romer Labs (Tulln, Austria). Monacolin K (25 mg), monacolin K acid (5 mg) and simvastatin (10 mg) were purchased from Vinci Biochem S.R.L. (Florence, Italy).

HPLC-grade ethanol, and acetonitrile were purchased from Sigma-Aldrich (Taufkirchen, Germany); bidistilled water was obtained using Milli-Q System (Millipore, Bedford, MA, USA). MS-grade formic acid from Fisher Chemical (Thermo Fisher Scientific Inc., San Jose, CA, USA) was also used.

### Red Yeast Rice Supplements: Sampling Plan

Thirty-seven red yeast rice food supplements, including pure RYR (8) and multi-ingredient formulations (26) with standardized amounts of monacolin K, were bought mainly on internet web sites (24) but also from local pharmacies in Italy (13) between September 2020 and February 2021. RYR products were formulated as capsules (18), tablets (15) and soft capsule (4). Four products were labeled as “citrinin free” (see “Note” column in Table 1). Detailed information on all samples with their form, suppliers, RYR daily dose and MKA content are listed in Table 1. All the samples were analyzed before their expiration date with UHPLC-MS/MS technique.

**Table 1:** List of supplements containing red yeast rice.

**N°**	**Formulation**	**Daily dose** **(Cps)**	**RYR per daily** **dose (mg)**	**Monacolin K declared** **on label (mg)**	**Supplier**	**Form**	**Notes**
1	Multicomponent	1	200	10	Pharmacy	Tablet	
2	Multicomponent	2	333.4	10	Pharmacy	Capsule	
3	Pure RYR	2	N.D.	10	On-line pharmacy	Capsule	
4	Pure RYR	2	200	10	On-line store	Capsule	
5	Pure RYR	1	600	4.8	On-line store	Capsule	
6	Multicomponent	1	250	10	On-line store	Capsule	Citrinin free
7	Pure RYR	1	300	12	On-line pharmacy	Capsule	Citrinin free
8	Multicomponent	1	210	10.5	On-line pharmacy	Capsule	
9	Multicomponent	1	600	N.D.	On-line pharmacy	Capsule	Citrinin free
10	Pure RYR	1	150	4.5	On-line store	Tablet	
11	Multicomponent	1	200	10	On-line store	Tablet	
12	Multicomponent	2	1,200	N.D.	On-line store	Capsule	
13	Multicomponent	1	100	3	Pharmacy	Tablet	
14	Multicomponent	1	200	10	On-line store	Capsule	
15	Multicomponent	1	200	10	On-line store	Capsule	
16	Multicomponent	1	100	3	On-line store	Capsule	
17	Multicomponent	1	200	10	On-line store	Capsule	
18	Pure RYR	1	200	10	Pharmacy	Tablet	
19	Pure RYR	2	1,200	N.D.	On-line store	Capsule	
20	Multicomponent	2	200	10	On-line store	Soft capsules	
21	Multicomponent	1	200	10	Pharmacy	Tablet	
22	Multicomponent	2	666.7	10	On-line store	Capsule	
23	Multicomponent	1	200	10	On-line store	Tablet	Citrinin free
24	Multicomponent	1	200	10	On-line store	Tablet	
25	Multicomponent	1	333.4	10	On-line store	Tablet	
26	Multicomponent	1	333	10	On-line store	Capsule	
27	Multicomponent	1	333	10	On-line store	Capsule	
28	Multicomponent	1	200	3	Pharmacy	Tablet	
29	Multicomponent	1	200	10	Pharmacy	Tablet	
30	Multicomponent	1	200	10	Pharmacy	Soft capsules	
31	Multicomponent	1	370.4	10	Pharmacy	Tablet	
32	Multicomponent	1	400	5	On-line store	Soft capsules	
33	Multicomponent	1	167	5	On-line store	Tablet	
34	Multicomponent	1	333	10	Pharmacy	Tablet	
35	Pure RYR	2	338.4	10	Pharmacy	Capsule	
36	Multicomponent	1	220	3.3	On-line store	Tablet	
37	Multicomponent	1	417	10	On-line store	Soft capsules	

*N.D., not declared on label*.

* *Statement made on label by the producer*.

### Sample Preparation for Mycotoxins Analysis

Tablets were weighed and then pulverized with a mortar and pestle. For capsules, five samples were weighed, opened and the contents mixed and triturated in a mortar and pestle. Soft capsules were punctured, and the liquid content was carefully squeezed into a beaker and weighed.

Sample preparation was performed following the protocol previously optimized by Wang et al., 2014 (27). To put it briefly, one gram of each sample was extracted with 30 ml ethanol/water (70:30) by shaking on a rotary shaker at 200 strokes/min for 30 min, and it was treated with ultrasonication at 40 °C for 30 min, and then shaken on a rotary shaker at 200 rpm for 1.5 h (27). The extract was centrifuged for 10 min at 1,4000 rpm at room temperature and then supernatants were quantitatively analyzed using LC-MS/MS.

### Targeted UHPLC-MS/MS Analysis of Mycotoxins and Monacolin K

UHPLC Dionex Ultimate 3000 separation system coupled to a triple quadrupole mass spectrometer (TSQ Vantage; Thermo Fisher Scientific Inc., San Jose, CA, USA) equipped with an electrospray source (ESI) was employed.

For the chromatographic separation, a reversed-phase C18 Kinetex EVO column (Phenomenex, Torrance, CA, USA) with 2.10 × 100 mm and a particle size of 2.6μm heated to 40 °C was used. 3 μl of sample extract was injected into the system; the flow rate was 0.35 ml/min.

Gradient elution was performed by using water (eluent A) and methanol (eluent B) both acidified with 0.1% formic acid. Initial conditions were set at 30% B followed by a linear change to 90% B in 5.5 min. 100% B was reached in 2.5 min and kept for 2 min, followed by a reconditioning step for 5 min using initial composition of mobile phases. The total run time was 16 min.

MS parameters: the ESI source was operated in positive ionization mode (ESI^+^); spray voltage 3,000 V, capillary temperature at 270 °C, vaporizer temperature was kept at 200 °C, sheath gas flow was set at 40 units and the auxiliary gas flow at 5 units. S-Lens RF amplitude value and collision energies (CE) were optimized during infusion of analyte standard solutions (1000 ng mL^−1^, in methanol) employing an automatic function of X-calibur software (Thermo Fisher Scientific, San Jose, CA, USA). Detection was performed using multiple reaction monitoring (MRM) mode. The following optimized transitions were used for the quantification: CIT m/z 251→ 233 (CE 18); OTA m/z 404→ 238 (CE = 21 eV); AFB1 *m/z* 317→ 285 (CE = 42 eV); AFB2 *m/z* 315→ 287 (CE = 25 eV); AFG1 *m/z* 329→ 243 (CE = 25 eV); AFG2 *m/z* 331→ 313 (CE = 30 eV); AFM1 *m/z* 329→ 273 (CE = 25 eV); MK *m/z* 427→ 335 (CE = 15 eV); MKA *m/z* 445→ 343 (CE = 20 eV); simvastatin *m/z* 441→ 325 (CE = 24 eV). Quantification of target analytes was performed using calibration standards (range 0.001–2 ppm for CIT, 1–50 ppm for MKA, 0.0125–1.25 ppm for simvastatin, and 5–100 ppm for MK). Further analytical details are summarized in Supplementary Table S1. Quantification of targeted analytics was performed employing Thermo Xcalibur 2.2.SP1 QuanBrowser software.

### Risk Dietary Exposure

For each RYR sample, the daily citrinin intake was established based on the suggested dosage and the mean capsule weight (measured in triplicate) and expressed as μg/kg b.w. considering a man of 60 kg. The Margin of Exposure (MOE) was calculated for each supplement, according to the guidelines previously reported (28).

## Results and Discussion

The aim of this study was to estimate whether RYR supplements comply with pharmaceutical quality, and whether their composition is consistent with label statements and with normative modifications related to maximum citrinin content introduced in the EU in April 2020. After setting up a dedicated extraction and LC-MS/MS detection protocol, 37 different food supplements were evaluated. The pool comprised both capsules of pure RYR or multi-ingredient formulations with standardized amounts of MK and other phytochemicals (see Table 1). Multiple endpoints were evaluated, namely monacolin content, potential adulteration, presence of synthetic statins and citrinin content. Data are reported in Tables 2, 3.

**Table 2:** Monacolins and simvastatin content of 37 RYR food supplements.

**Sample**	**Type**	**MK**	**MKA**	**MK+MKA**	**Label vs.** **content, difference**	**MKA/MK+MKA**	**Simvastatin**
		**mg/daily dose**		**mg/daily dose**			**μg/daily dose**
1	FS	10.6 ± 1.5	3.0 ± 1.3	13.6	36%	0.35	1.2 ± 0.4
2	RYR	24.6 ± 0.2	2.0 ± 0.3	26.6	166%	0.25	4.9 ± 0.9
3	RYR	27.7 ± 0.8	1.6 ± 0.1	29.3	193%	0.24	1.5 ± 0.0
4	RYR	1.0 ± 0.9	3.6 ± 0.1	4.6	−46%	0.64	0.1 ± 0.0
5	RYR	1.6 ± 0.4	1.9 ± 0.0	3.5	−27%	0.48	0.2 ± 0.0
6	RYR	0.5 ± 0.005	3.3 ± 0.6	3.8	−62%	0.37	1.2 ± 0.0
7	RYR	27.3 ± 0.6	0.4 ± 0.1	27.7	131%	0.13	2.1 ± 0.0
8	FS	11.2 ± 1.2	0.3 ± 0.0	11.5	9.50%	0.11	2.2 ± 0.0
9	RYR	1.2 ± 0.03	< LOQ	1.2	n.a.	0.03	< LOQ
10	RYR	6.7 ± 0.2	1.2 ± 0.0	7.9	76%	0.37	1.1 ± 0.0
11	FS	6.8 ± 0.7	0.7 ± 0.0	7.4	−36%	0.17	3.4 ± 0.0
12	RYR	2.8 ± 0.1	1.2 ± 0.0	4.0	n.a.	0.22	0.8 ± 0.0
13	FS	7.7 ± 0.2	0.5 ± 0.0	8.2	173%	0.18	1.0 ± 0.0
14	FS	12.4 ± 0.02	0.8 ± 0.0	13.2	32%	0.23	3.8 ± 0.0
15	FS	6.1 ± 3.0	0.7 ± 0.0	6.8	38%	0.25	1.5 ± 0.0
16	FS	1.6 ± 0.4	0.1 ± 0.0	1.7	−83%	0.06	1.4 ± 0.1
17	FS	27.1 ± 0.6	0.5 ± 0.0	27.6	176%	0.14	2.0 ± 0.0
18	RYR	< LOQ	< LOQ	0.0	n.a.	0.11	< LOQ
19	FS	34.7 ± 3.7	1.3 ± 0.0	36.0	266%	0.21	7.5 ± 0.2
20	FS	9.0 ± 2.5	3.3 ± 0.9	12.3	23%	0.40	1.2 ± 0.0
21	FS	16.1 ± 0.8	1.8 ± 0.0	17.9	79%	0.26	4.9 ± 0.1
22	RYR	11.4 ± 3.4	1.0 ± 0.0	12.4	24%	0.27	1.9 ± 0.0
23	FS	7.3 ± 0.2	< LOQ	7.3	−27%	0.41	< LOQ
24	FS	16.5 ± 1.3	1.5 ± 0.0	18.0	80%	0.23	1.4 ± 0.0
25	FS	5.9 ± 0.2	0.7 ± 0.0	6.6	−34%	0.20	1.9 ± 0.0
26	FS	5.8 ± 1.4	1.1 ± 0.1	6.9	39%	0.25	1.6 ± 0.0
27	FS	17.4 ± 1.6	1.2 ± 0.1	18.6	86%	0.24	1.6 ± 0.0
28	FS	20.1 ± 1.2	1.0 ± 0.2	21.1	111%	0.18	5.1 ± 0.1
29	FS	18.5 ± 0.4	0.7 ± 0.0	19.2	92%	0.21	1.8 ± 0.0
30	FS	10.0 ± 1.5	3.3 ± 1.3	13.3	33%	0.26	5.3 ± 0.2
31	FS	3.1 ± 0.6	1.4 ± 0.1	4.5	10%	0.20	2.3 ± 0.0
32	FS	22.6 ± 0.1	1.4 ± 0.1	24.0	140%	0.28	2.9 ± 0.1
33	RYR	18.3 ± 0.3	1.3 ± 0.3	19.6	96%	0.24	3.2 ± 0.1
34	FS	1.3 ± 0.0	0.9 ± 0.0	2.2	−33%	0.24	0.9 ± 0.0
35	FS	18.5 ± 0.1	< LOQ	18.5	85%	0.19	< LOQ
36	FS	11.8 ± 0.01	< LOQ	11.8	18%	0.21	< LOQ
37	FS	5.2 ± 0.02	0.5 ± 0.2	5.7	−43%	0.15	1.8 ± 0.1

*LOQ, limit of quantification; n.a., data not available*.

**Table 3:** Citrinin content and MOE determination for 37 RYR food supplements.

**Sample**	**Citrinin**			
	**μg/g**	**μg/daily dose**	ug/kg bw^*^	**MOE**
1	4.7 ± 0.2	1.97	0.033	608
2	8.8 ± 2.0	8.27	0.138	145
3	2.3 ± 0.02	2.07	0.035	580
4	1.9 ± 0.1	1.63	0.027	734
5	3.2 ± 0.1	2.43	0.041	493
6	4.1 ± 0.2	2.05	0.034	585
7	2.5 ± 0.0	1.23	0.020	980
8	2.5 ± 0.2	1.35	0.023	889
9	3.2 ± 0.05	2.27	0.038	528
10	2.6 ± 0.3	1.30	0.022	923
11	8.7 ± 0.2	4.79	0.080	251
12	3.0 ± 0.1	4.98	0.083	241
13	3.4 ± 0.01	1.87	0.031	642
14	12.3 ± 0.7	5.66	0.094	212
15	4.3 ± 0.3	2.28	0.038	527
16	2.1 ± 0.04	1.18	0.020	1,020
17	0.1 ± 0.1	0.03	0.001	36,364
18	4.1 ± 0.1	6.15	0.103	195
19	5.4 ± 0.2	8.21	0.137	146
20	4.5 ± 0.1	2.43	0.041	494
21	25.1 ± 1.8	23.09	0.385	52
22	9.2 ± 0.2	4.97	0.083	242
23	7.3 ± 0.1	3.80	0.063	316
24	1.1 ± 0.04	1.23	0.021	974
25	8.2 ± 0.3	4.10	0.068	293
26	4.8 ± 0.001	4.80	0.080	250
27	10.7 ± 0.5	8.35	0.139	144
28	8.8 ± 0.2	6.86	0.114	175
29	8.0 ± 0.4	6.48	0.108	185
30	0.9 ± 0.1	3.47	0.058	345
31	3.2 ± 0.7	4.29	0.071	280
32	8.7 ± 0.07	5.66	0.094	212
33	4.8 ± 2.2	4.22	0.070	284
34	2.9 ± 0.2	2.90	0.048	414
35	5.7 ± 0.6	4.79	0.080	251
36	13 ± 0.2	5.98	0.100	201
37	7.2 ± 0.6	3.60	0.060	333

* *Considering a reference body weight for human of 60 kg [see ref. #(29)]*.

### Compliance Between Label and Actual MK Content

**Figure 1:**
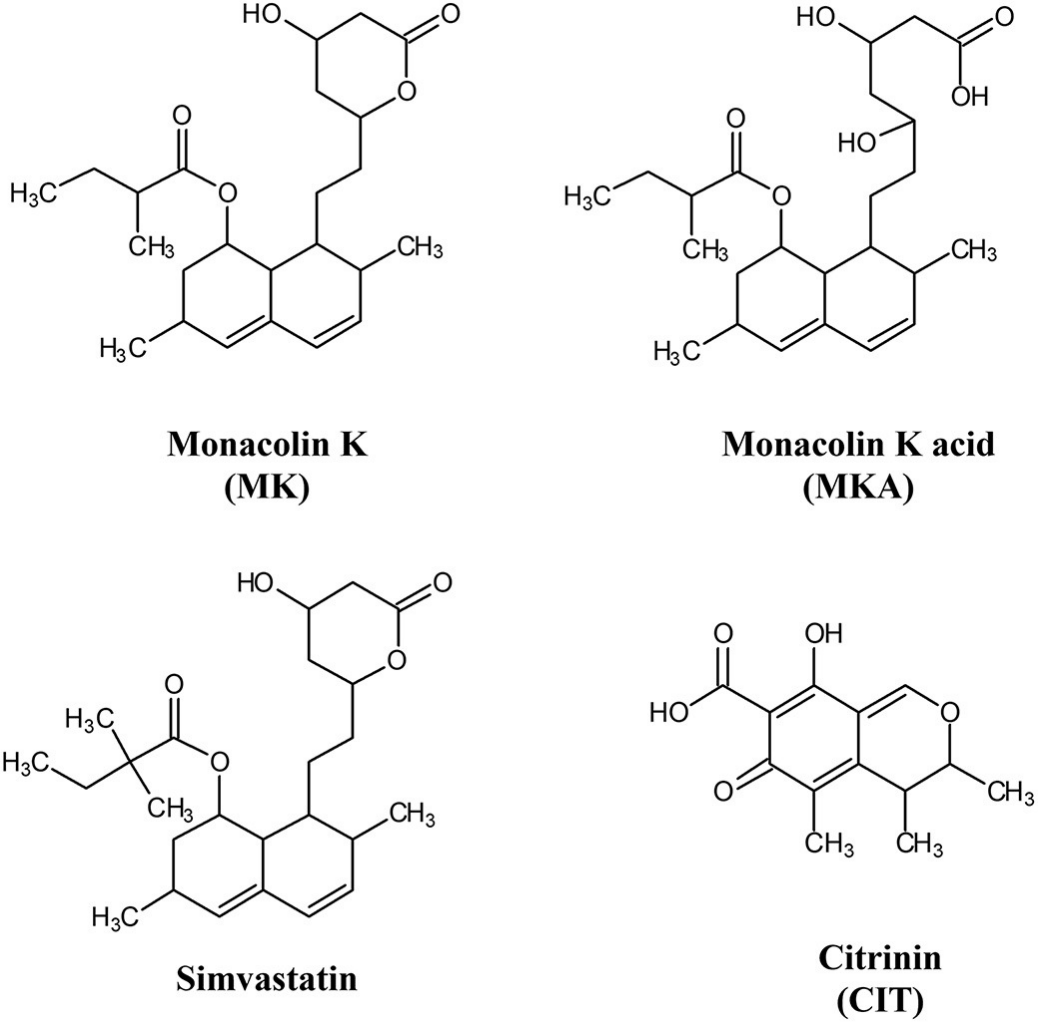
Chemical structures of monacolin K (MK), monacolin K acid (MKA), simvastatin and citrinin (CIT).

A first evaluation concerned whether some pharmaceutical quality criteria can be applied to food supplements. At present, no standardization is required in the EU, while EFSA dictate that a daily intake of 10 mg of MK is mandatory to obtain the effects related to RYR health claim awarded in 2011. During the fermentation process, the form responsible for the inhibition of cholesterol synthesis (MKA) prevails, favored by water solubility and slightly acidic pH, with a content of approximately 80% on average. The final, drying step in RYR production induces a partial cyclization of MKA. Therefore, in the finished product, the acid: lactone ratio usually varies from 6: 4 to 4:6. The lactone form (MK) can be hydrolyzed into MKA *in vivo* by the action of hydroxyl esterase (see Figure 1).

To provide an unequivocal determination, calculations were therefore made according to total monacolin K content, i.e. considering both lactone (MK) and acidic form (MKA). Data reported in Table 2 confirm that MK is not standardized in food supplements at present available in both retail and online marketplaces, with major relapses in terms of efficacy, safety and exposure. Nine products provided a lower content as compared to values declared on labels (from −30 to −83%), 24 had a larger MK amount (from 10 to 266%), and three products had negligible MK contents. The actual MK dosage was highly variable, exceeding a 30-fold difference from 1.2 to 36 mg/day and confirming a trend emerged during a recent survey on 26 brands of RYR from large retail chains in the United States (30). No sample had label values compliant with pharmaceutical Good Manufacturing Practices (GMP) requirements (95–105% content of active constituent). In 2011 a health claim has been awarded to RYR by EFSA, stating that “Monacolin K from RYR contributes to the maintenance of normal blood cholesterol concentrations”. Such claim boosted sales of the ingredient and is supported by a large scientific evidence in multiple clinical trials. In order to obtain the claimed effect, according to EFSA 10 mg of monacolin K from fermented RYR preparations should be consumed daily and therefore most producers state to provide such amount. According to our data, however, only five brands provide a content in an arbitrary range comprised between 8 and 12 mg/die.

Monacolin K biosynthesis is reportedly variable according to multiple factors, including strain, temperature, fermentation techniques and parameters that may lead to an unsatisfactory content in *Monascus* fermentations (26, 31). Precisely for this reason, standardization and post-production control should be extremely accurate, in order to guarantee the same dosage to any consumer and limit the risk of side effects due to excessive administration. The lack of standardization reported represents instead a major shortcoming, outlining a difficult translation from evidence-based science to commercial reality, as clinical and nutritional trials carried out with pure and carefully weighed compounds may not be replicated at post-marketing level. The same critical point concerns the way experimental protocols are set up in those clinical trials using actual supplements instead of formulation prepared (and dosed) on purpose (32). On this regard, a strong commitment to careful analytical determination of MK should be reiterated for any trial intended to test the efficacy of commercial food supplements. As other authors have suggested, results from trials in which an accurate standardization of RYR supplements are not performed may result in unreliable and contradictory data (18, 30). The same applies when exposure is determined to estimate adverse effects and their incidence in the general population: as lipid lowering effects, also most common side effects of RYR are related to MK content and a higher or lower incidence or the emergence of case reports may be ultimately the result of inappropriate dosage stemming from poor standardization, as the 10 products exceeding 90% of declared MK seem to suggest. The trend toward excess is particularly relevant especially considering that the MK contained in the RYR would have a higher bioavailability than the drug to the point that, according to a study (33), 5–6 mg would be equivalent to 20–40 mg of pure, pharmaceutical-grade lovastatin.

### Potential Adulteration

Nannoni et al. found that monacolin K and its acidic form have a proportional relationship, which can be used to determine whether commercial lovastatin has been added to the RYR (34).

By applying the same formula to our data (Table 2), we found that the ratio between the MK / MK + MKA areas is lower than 0.3 in 29 samples, meaning that 80% of the total may be adulterated by the addition of MK from external sources, likely in the form of lovastatin produced by *Aspergillus terreus* or by other means (18, 35). Variable, but limited, amounts of simvastatin (0.1–7.5 μg per daily dose) were also found in 30 samples (Table 2). While these amounts are of no concern, such evidence may open interesting perspectives, as simvastatin is reportedly a synthetic statin. Further investigations may disclose its origin, whether these trace amounts are a consequence of some kind of adulteration (e.g. an impurity from the addition of foreign material) or the result of a previously unreported synthetic capability of *Monascus purpureus*. In both cases the results would be noteworthy, as simvastatin is a derivative of lovastatin whose production is a multistep process. A biocatalytic process starting from monacolin J and a fermentative process for biotransformation of lovastatin into simvastatin, based on *Aspergillus terreus* ATCC20542 fed with 2,2- dimethylbutyrate are available (36).

### Mycotoxin Content

Since 2017 EFSA clearly identified RYR as the food product with the highest incidence of citrinin contamination (22). Multiple investigations reported frequent and sometimes alarming amounts of this mycotoxin in RYR and our data confirm this evidence. For instance, these data are agreement with previous surveys, in which citrinin was quantified in RYR ranging from 10 to 44240 μg/kg (21, 37, 38). By comparing these literature data with the citrinin levels collected by our study, it can be confirmed that citrinin is almost always present in red rice supplement samples, with values often higher than the legal limit. All tested samples had in fact a citrinin content above LOQ, with values ranging from 100 to 25100 μg/Kg, translated in daily dosages comprised between 0.05 and 23.1 μg (Table 3). Four products were compliant with maximum citrinin levels in force in the EU until April 2020, while only a single product was compliant with the limit of 100 μg/kg introduced after that date. Four contaminated products were labeled as “citrinin free”, posing worries of the relevance of this unregulated, self-declared claim which, besides its actual unreliability, seems also to deceptively suggest that products otherwise labeled are of inferior quality.

No ochratoxins nor aflatoxin B1, B2, G1, G2 or M1 were detected in neither sample. However, in 29 samples, an isomeric form of ochratoxin A (OTA) was found. The latter and OTA shown identical fragmentation patterns (see Supplementary Figure S1, Supplemental files), making a mass spectrometric differentiation impossible. On the other hands, the later chromatographic elution (9.7 min) of such isomeric form from the C18 column compared with OTA standard (7.4 min) is consistent with the putative annotation as 2′R-ochratoxin A (2′R-OTA) (see Supplementary Figure S1, Supplemental files). Cramer and co-authors (39) previously reported the identification of 2′R-OTA as the thermal degradation product of OTA in coffee. Its presence, if confirmed with the analytical standard, suggests that OTA may be produced by *Monascus purpureus* or more likely inherited by rice during fermentation after and then subject to degradation during the drying step. This modified form of OTA has a more than seven-fold higher biological half-life in human blood compared to parent compound and while amounts are negligible from toxicological standpoint, its presence suggest that careful, wide-spectrum mycotoxin monitoring could be advisable for RYR.

EFSA has set a level of no concern for citrinin-caused nephrotoxicity at 0.2 μg/kg body weight per day, but it has acknowledged the need for more reliable data on the citrinin level in food and feed before it can undertake exposure studies and risk assessment (40).

Our survey suggests that such reliability, given the wide range of contamination detected, is difficult to obtain.

### Exposure Assessment to Citrinin

Due to the use of RYR as a natural alternative to statins in lipid-lowering control, it is usually consumed on a regular basis for a long period of time. Therefore, the exposure to possible contaminants should be regarded as chronic.

Based on the scientific evidence, EFSA has classified citrinin as a genotoxic compound. Accordingly, the assessment of possible safety concerns arising from its presence in food and feed, should follow a margin of exposure (MOE) approach. The MOE is the ratio of no-observed-adverse-effect level (NOAEL) obtained from animal toxicology studies to the estimated human exposure level. Under this approach, a MOE ≥ 10,000 is considered of no concern for genotoxic and carcinogenic compounds (28). Although usually for genotoxic compounds a benchmark dose (BMD) approach is usually preferred for the derivation of health-based guidance value, in the case of citrinin only a level of no concern of 0.2 μg/kg b.w. per day is available so far (41). This approximation might lead to an underestimation of the associated risk, being the NOAEL less conservative than the benchmark dose.

The exposure assessment was calculated for all the considered samples, based on a reference body weight for human of 60 kg (29), and reported in Table 3. It can be noticed that in 34 out of 37 samples, the daily intake exceeds the NOAEL, with a maximum of 0.385 μg/kg b.w. for sample #21. When it comes to the MOE, the values are far from the safety threshold of 10,000 for all the batches, with the only exception of sample #17.

Although largely cautionary, the MOE approach clearly shows that the exposure to citrinin from RYR supplements may pose a risk for the consumers. Besides arguments related to public health and regulatory measures, our outcome is even more relevant in terms of communication, when considering that a “citrinin-free” claim was reported on the label of four batches samples in this study. The halo of naturality of RYR supplements together with the associated “claims”, may prompt the consumers to subjectively increase the daily dosage without any perception of possible risk. Therefore, such unsolicited claims should be strongly mistrusted and carefully regulated.

It must be noticed that, compared to food the intake of RYR supplements is less affected by variability ad a consequence of their pre-dosed form. Users, often prompted by health-related issues, take supplements on a regular base according to the suggested dosage, and often choose the same brand over time. While the exposure assessment to contaminants may vary a lot between mean and high-consumers of a certain food, in the case of RYR supplements the variability – and therefore the associated uncertainty – is strongly reduced. As some recent investigations have noticed variability the urinary excretion of citrinin as a marker to assess the exposure, it would be advisable if similar research in the future could track RYR users within the screened population (42).

Considering the toxicological relevance of citrinin, our data poses a serious health concern, and once again underline the need of quality standard and control plan for food supplements.

Overall, our investigation confirms that quality of RYR food supplements is dubious and strict quality control should be implemented. Most commercial RYR products do not meet the maximum level of citrinin allowed in the EU starting April 2020. To date, (accessed 9 January 2021) RASFF database reports only 9 notifications regarding fermented red rice supplements, starting since 2010. Three were related to citrinin content and one to an excess of monacolin; given the occurrence reported in our and in other recently published papers for both these substances, post marketing controls vastly underestimate the scale of the problem. Therefore, pharmaceutical quality criteria should not be taken for granted by both researchers, clinicians, regulators, producers and consumers dealing with RYR. More rigorous standardization and monitoring for mycotoxin content must be encouraged to extrapolate data for risk assessment with respect to the prevalence of citrinin. Given the widespread use, the equivalence between MK and lovastatin and the occurrence of side effects, for RYR supplements the enforcement of pharmaceutical GMP could be a response in order to mitigate the present situation.

## Data Availability Statement

The raw data supporting the conclusions of this article will be made available by the authors, without undue reservation.

## Author Contributions

LR, RB, and CD'A conceived the study. LR was responsible for the sampling plan, planned and carried out the LC-MS analysis, and the data elaboration. RB and CD'A contributed to the interpretation of the analytical results. RB supervised the project. All authors contributed to the writing process and critically revised the final manuscript.

## Conflict of Interest

The authors declare that the research was conducted in the absence of any commercial or financial relationships that could be construed as a potential conflict of interest.

## Publisher's Note

All claims expressed in this article are solely those of the authors and do not necessarily represent those of their affiliated organizations, or those of the publisher, the editors and the reviewers. Any product that may be evaluated in this article, or claim that may be made by its manufacturer, is not guaranteed or endorsed by the publisher.
