# Supplementary material for: Risk Assessment of RYR Food Supplements: Perception vs. Reality
Source: Front Nutr. 2021 Dec 7;8:792529. doi: 10.3389/fnut.2021.792529 (PMC8691514; doi:10.3389/fnut.2021.792529)
Supplement: Supplementary file 1 [file Data_Sheet_1.pdf]

Supplementary Material

Perception versus reality: multiple issues affect quality in RYR food supplements.

Laura Righetti*, Chiara Dall'Asta*, Renato Bruni

Department of Food and Drug, University of Parma, Viale delle Scienze 17/A, I-43124 Parma, Italy

*Corresponding author

Dr. Laura Righetti, laura.righetti@unipr.it

Prof. Chaira Dall'Asta, chiara.righetti@unipr.it

Supplementary Table 1. Main in-house validation parameters.

	**CIT**	**MK**	**MKA**	**Simvastatina**
Calibration range (mg/kg) [a]	0.001-2	5-100	1-50	0.0125-1.25
LOQ (µg/kg)	1	5000	1000	12.5
LOD (µg/kg)	0.001	0.1	1	0.01

[a] High linearity (R2>0.99) has been observed in the used calibration.

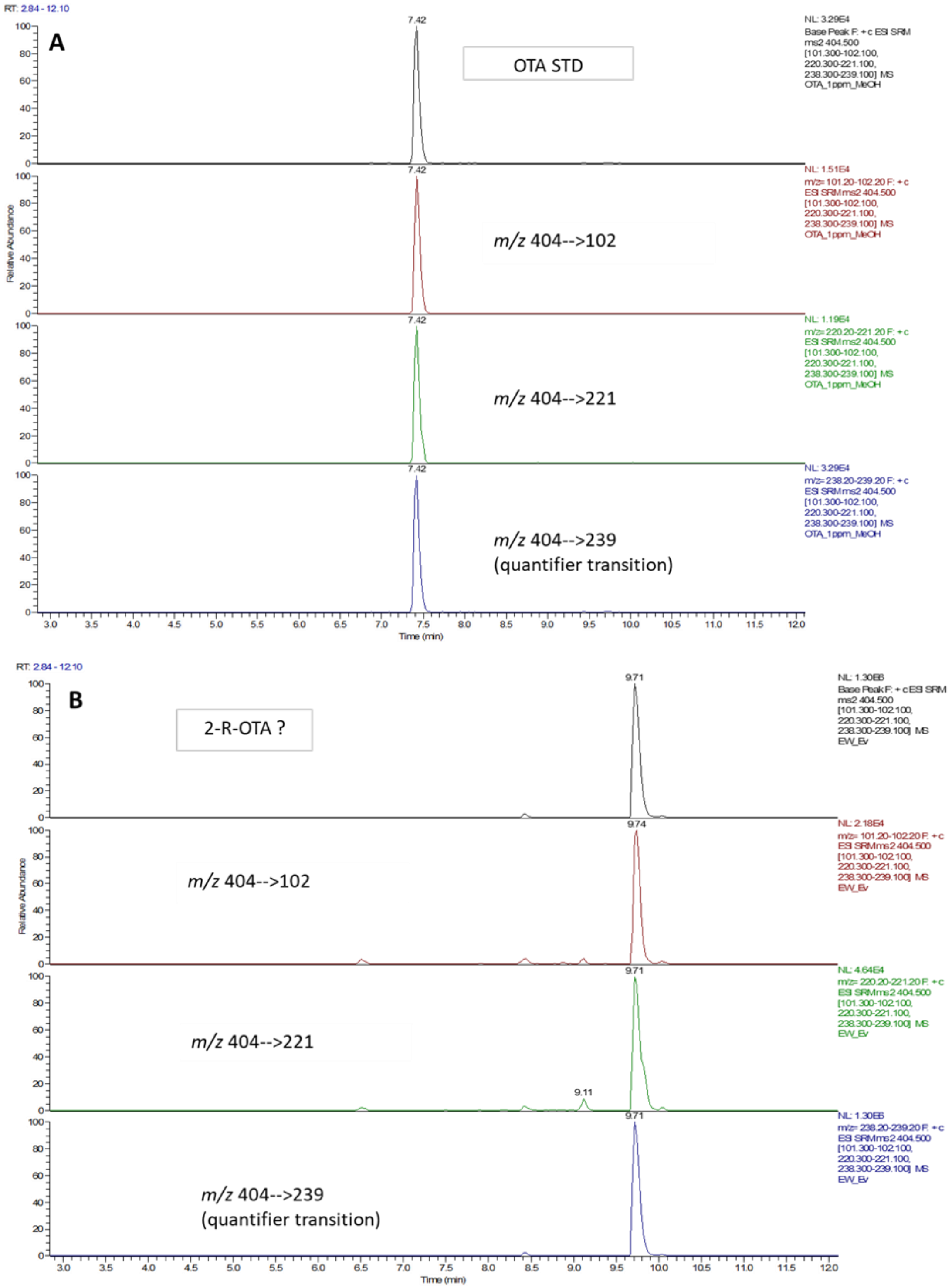

Supplementary Figure 1. Extracted ion chromatograms and fragmentation pattern of OTA and its putatively annotate metabolite. (A) LC-MS/MS spectrum of (A) protonated standard of OTA and (B) putatively annotated 2'R-ochratoxin A in RYR sample extract, showing identical fragmentation patterns. MRM transitions *m/z* 404 → *m/z* 239 yielded the best sensitivity for both compounds.
